# Anti-PM/Scl antibodies are found in Japanese patients with various systemic autoimmune conditions besides myositis and scleroderma

**DOI:** 10.1186/s13075-015-0573-x

**Published:** 2015-03-11

**Authors:** Yoshinao Muro, Yuji Hosono, Kazumitsu Sugiura, Yasushi Ogawa, Tsuneyo Mimori, Masashi Akiyama

**Affiliations:** Department of Dermatology, Nagoya University Graduate School of Medicine, 65 Tsurumai-cho, Showa-ku, Nagoya, 466-8550 Japan; Department of Rheumatology and Clinical Immunology, Kyoto University Graduate School of Medicine, Sakyo-ku, Kyoto, 606-8507 Japan; Division of Connective Tissue Disease and Autoimmunity, Department of Dermatology, Nagoya University Graduate School of Medicine, 65 Tsurumai-cho, Showa-ku, Nagoya, 466-8550 Japan

## Abstract

**Introduction:**

Anti-PM/Scl antibodies are associated with polymyositis (PM)/systemic scleroderma (SSc) overlap syndromes and are also found in other systemic autoimmune diseases. Although anti-PM/Scl reactivity is found in 3-11% of PM or SSc patients and in approximately 25% of PM/SSc overlap patients, previous large studies of Japanese patients with scleroderma reported that anti-PM/Scl are not found in Japanese patients at all. The PM/Scl autoantigen complex comprises 11–16 different polypeptides; ELISA with PM1-α peptide, which is a major epitope of the PM/Scl complex, has frequently been used for the detection of these antibodies in recent studies. However, no ELISA kit is commercially available in Japan.

**Methods:**

In this study, we developed an immunoassay for measuring antibodies against recombinant PM/Scl-100 and PM/Scl-75 polypeptides, which are the two major targets of the complex, and we investigated their presence in 600 Japanese patients with various systemic autoimmune conditions. Immunoprecipitation analysis using the recombinants in addition to traditional radiolabeled cell extracts were also applied to ELISA-positive sera.

**Results:**

In ELISA, 11 patients were positive for anti-PM/Scl-100 antibodies and 7 of these 11 patients were also positive for anti-PM/Scl-75 antibodies. Immunoprecipitation analysis using the recombinants in addition to traditional radiolabeled cell extracts confirmed that 9 out of these 11 patients immunoprecipitated the typical sets of PM/Scl proteins. In total, 4/16 (25%) undifferentiated connective tissue disease (UCTD) patients, 3/126 (2.4%) dermatomyositis patients, 1/223 (0.4%) SSc patients, 1/88 (1.1%) Sjögren’s syndrome patients, 0/123 patients with systemic lupus erythematosus, 0/17 patients with overlap syndrome and 0/7 patients with PM were judged to be positive for anti-PM/Scl antibodies.

**Conclusions:**

This is the first report of Japanese autoimmune patients with anti-PM/Scl antibodies. In Japanese patients, anti-PM/Scl antibodies are only very rarely found, and they are not always specific for dermatomyositis (DM) or SSc; they are also present in various autoimmune conditions with the highest prevalence being in UCTD. All anti-PM/Scl-positive DM cases are complicated with interstitial lung disease and/or cancer, while no life-threatening involvement was found in other anti-PM/Scl-positive cases. Further studies on larger cohorts are necessary to define the clinical significance of anti-PM/Scl antibodies in autoimmune diseases.

## Introduction

A characteristic feature of patients with systemic autoimmune diseases is the presence of autoantibodies in their sera that target intracellular components [1]. Some of these autoantibodies are useful diagnostic markers for various systemic autoimmune diseases [1-3]. Some autoantibodies have great diversity in their prevalence among different races and countries [4-6].

Anti-PM/Scl antibodies, first described as ‘anti-PM-1’ in 1977, were found in patients with overlap syndrome of polymyositis (PM) and scleroderma (Scl) [7]. Anti-PM/Scl antibodies produce a homogenous nucleolar pattern in indirect immunofluorescence (IIF) staining and recognize the PM/Scl complex, which is the human counterpart of the yeast exosome and consists of 11 to 16 polypeptides [8]. Most anti-PM/Scl antibodies recognize two components, PM/Scl-100 and PM/Scl-75 [9-11], and are found mostly in patients with overlap syndrome (OL) of PM and systemic scleroderma (SSc) (approximately 25%) [12], as well as in PM or SSc patients (3% to 13%) [13]; however, they are rarely found in other diseases, such as Sjögren’s syndrome (SS) [14]. For the detection of anti-PM/Scl antibodies, several techniques have been utilized: double immunodiffusion, immunoprecipitation (IPP), enzyme-linked immunosorbent assay (ELISA) and line immunoassay (LIA) [15]. ELISA using the PM-1α synthetic peptide, a major epitope of PM/Scl-100 composed of an alpha helical structure located at amino acid 231 to 245 of PM/Scl-100 [16], was used in a recent multicenter study that elucidated the diagnostic and prognostic relevance of anti-PM/Scl antibodies in SSc clinics [17]. Unfortunately, this ELISA kit is not available in Japan.

The frequencies of some autoantibodies vary by ethnicity. For example, in a U.S. SSc cohort, in African-American patients, anti-U3-RNP (fibrillarin) antibodies were found in 30% of patients; meanwhile anti-Th/To antibodies were found in only 4% [4]. In white patients, however, anti-Th/To antibodies were found in 9%, whereas anti-U3-RNP antibodies were found in only 3% [4]. Another example is that anti-RNA polymerase III antibodies were less prevalent in French patients than in U.S. patients [7]. Although anti-PM/Scl antibodies are found in certain populations of patients in Western countries, as stated above, clinical studies on Japanese autoimmune patients to detect these antibodies have not been reported. Surprisingly, in two large SSc cohorts from two Japanese centers, no anti-PM/Scl-positive patients were found among 272 and 316 patients, respectively [18].

We recently developed a method that allows for the rapid conversion of cDNAs to a chemiluminescent ELISA to detect autoantibodies in human sera [19]. In this study, we constructed an ELISA for measuring anti-PM/Scl-100 and also anti-PM/Scl-75 antibodies, in order to screen these antibodies in 600 patients with various autoimmune conditions from a single center in Japan, and we investigated their clinical significance in Japanese patients.

## Methods

### Serum samples

Serum samples were collected from 600 Japanese patients, consisting of 223 with SSc, 126 with dermatomyositis (DM), 123 with systemic lupus erythematosus (SLE), 88 with SS, 17 with OL, 7 with PM and 16 with undifferentiated connective tissue disease (UCTD), between 1994 and 2014 at Nagoya University Hospital. SSc was diagnosed according to the classification of the American College of Rheumatology (ACR) [20] or the ACR/European League Against Rheumatism (EULAR) 2013 classification criteria [21]. Of the SSc patients, 185 were classified as diffuse cutaneous and 85 as limited cutaneous, according to the criteria of LeRoy and colleagues [22]. The DM patients (76 with adult DM, 12 with juvenile DM (JDM) and 38 with clinically amyopathic DM (CADM)) and PM patients fulfilled Bohan and Peter’s criteria [23], except for CADM, which was defined by Sontheimer’s criteria [24]. SLE was diagnosed by the ACR criteria for SLE [25]. SS was diagnosed based on Japanese diagnostic criteria [26]. OL, including 11 patients with PM + SSc, was diagnosed as cases that fulfilled the criteria for two systemic autoimmune diseases. UCTD was diagnosed according to the preliminary classification criteria proposed by Mosaca and colleagues [27]. Interstitial lung disease (ILD) was diagnosed by chest radiograph or chest computed tomography (CT) scan. Clinical information was collected retrospectively by reviewing their medical charts. Our cohort consisted of newly diagnosed incipient patients, except for a few patients with juvenile DM. As for patients with UCTD, serum samples were collected at the first visit. These patients were confirmed, by follow-up with doctors, as not fulfilling the criteria for defined CTD for at least three years from the beginning of symptoms according to the criteria of UCTD [27]. As control samples, serum samples from 72 healthy volunteers were also used. This study was conducted with the approval of the ethics committees of the Nagoya University Graduate School of Medicine and the Kyoto University Graduate School of Medical Science. All patients gave written consent to participate in the study.

### Recombinant antigens for ELISA and immunoprecipitation

The full-length cDNA clones of PM/Scl-100 (product No. FXC03779) and PM/Scl-75 (product No. FXC22044) were purchased from Flexi® ORF Clone (Promega, Madison, WI, USA). Biotinylated recombinant proteins were produced from the cDNA, using the T7 Quick Coupled Transcription/Translation System (Promega) according to our published protocol [28]. In short, 800 μl transcription and translation (TnT) Quick Master Mix, 20 μl 1 mM methionine, 30 μl transcend biotin-lysyl-tRNA, 120 μl water and 30 μl DNA (1 μg/μl) were mixed and then incubated at 30°C for 60 minutes.

### ELISA

**Figure 1:**
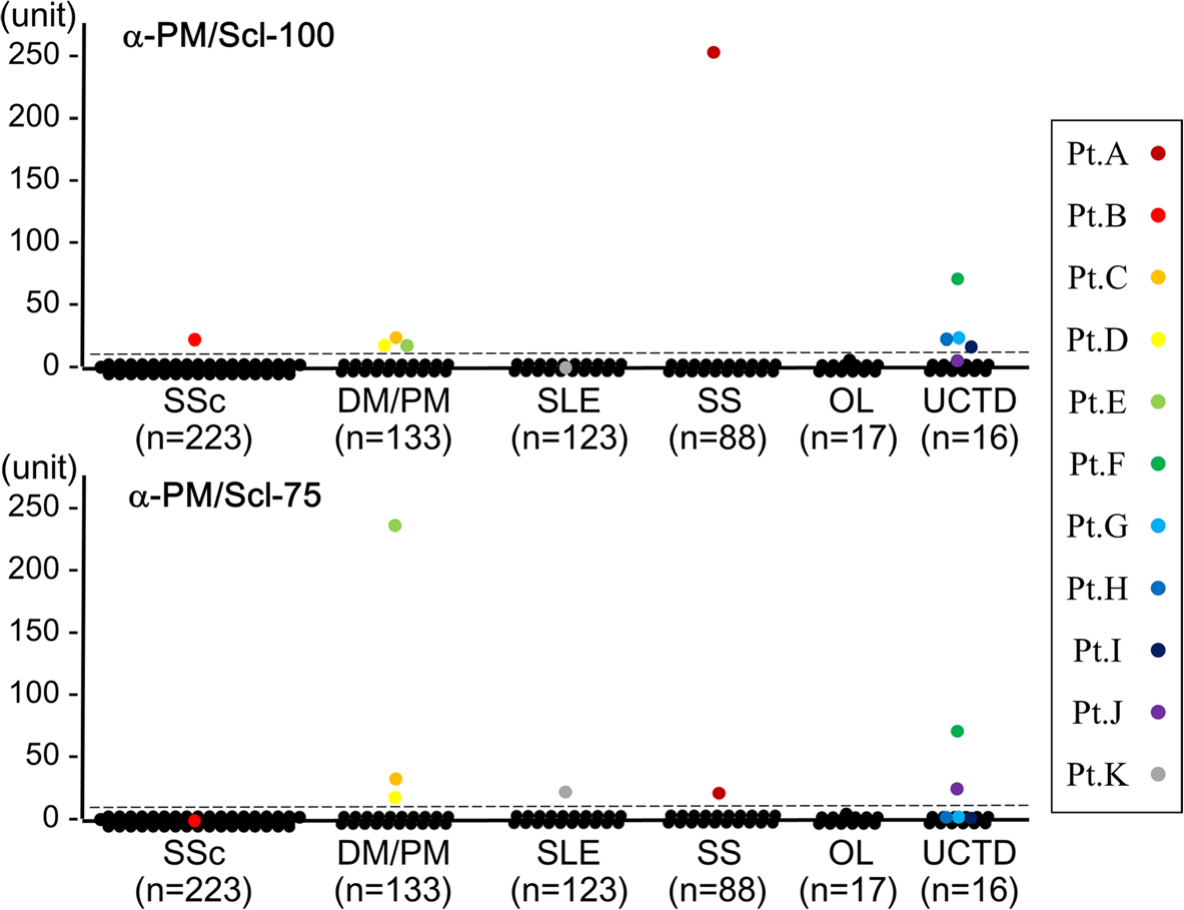
Qualitative measurement of anti-PM/Scl antibodies in ELISA. ELISA units of anti-PM/Scl-100 and anti-PM/Scl-75 antibodies are shown for a total of 600 serum samples from patients with various diseases. The antibody units are calculated from relative luminescence units using a standard curve obtained from serial concentrations of serum samples: patient A’s serum for anti-PM/Scl-100 ELISA and patient E’s serum for anti-PM/Scl-75 ELISA. The broken line indicates the cutoff value, which is the mean value of 36 healthy controls + 5 standard deviations. DM, dermatomyositis; OL, overlap syndrome; PM, polymyositis; SLE, systemic lupus erythematosus; SS, Sjögren’s syndrome; SSc, systemic scleroderma; UCTD, undifferntiated connective tissue disease.

Antibodies against PM/Scl-100 and PM/Scl-75 were tested by antigen-capture ELISA according to our published protocols [19]. Briefly, a 96-well Nunc™ Immobilizer™ Streptavidin Plate (Thermo Scientific Nunc, Roskilde, Denmark) was incubated with 1 μl/well of *in vitro* TnT reaction mixture including biotinylated recombinant protein. Wells were then incubated with 1:1000 diluted sera and probed with anti-human immunoglobulin G (IgG) antibody conjugated with horseradish peroxidase (HRP) (Dako, Glostrup, Denmark) (1:30,000 dilution). After incubation with SuperSignal® ELISA Femto Maximum Sensitivity Substrate (Thermo Scientific Pierce, Rockford, IL, USA), the relative luminescence unit (RLU) was determined using the GloMax®-Multi Detection System (Promega). Each serum sample was tested in duplicate, and the mean RLU with the background subtracted was used for data analysis. The RLU of the samples was converted into units using a standard curve created by a prototype positive serum. As a standard, the high-titer anti-PM/Scl-100 (patient A in Figure 1) or anti-PM/Scl-75 (patient E in Figure 1) antibody-positive sera diluted 1:5 serially, starting from 1:500, was run. Units correlated with the titers of antibodies: 1:500 dilution, 625 units; 1:2,500, 125 units; 1:12,500, 25 units; 1:62,500, 5 units; 1:312,500, 1 unit; 1:1,562,500, 0.2 units. The cutoff values (4.4 units for anti-PM/Scl-100 antibody and 2.1 units for anti-PM/Scl-75 antibody) were determined as the mean of the units obtained from 36 control sera from healthy volunteers + 5 standard deviations (SD).

### Immunoprecipitation

IPP was performed using TnT products as previously described [28] and using radiolabeled extracts of HeLa cells [29]. Prototype sera containing anti-PM/Scl, anti-MDA5, anti-TIF1γ or anti-Mi-2 antibodies from TM’s laboratory were also used.

### Laboratory tests and serological assay

Sera that were positive for anti-PM/Scl by ELISA were analyzed with an IIF laboratory kit using HEp-2 cells (Fluoro HEPANA Test; MBL, Nagoya, Japan) [30]. The samples were also screened by ELISA for antibodies against CCP, SS-A, SS-B, U1-RNP, Sm, CENP-B, ribosomal P, aminoacyl tRNA synthetase (ARS) and ds-DNA with commercial kits (MBL, Nagoya, Japan). This anti-SS-A kit detects only anti-SS-A/Ro60 and not anti-SS-A/Ro52/TRIM21.

### Statistical analyses

Data were statistically evaluated using SPSS Statistics (IBM, Tokyo, Japan). Fisher exact probability tests were used for comparison of frequencies. Mann–Whitney U tests were used for comparison of ELISA units. *P* values of less than 0.05 were considered significant.

## Results

### Measurement of anti-PM/Scl antibodies by ELISA

**Figure 2:**
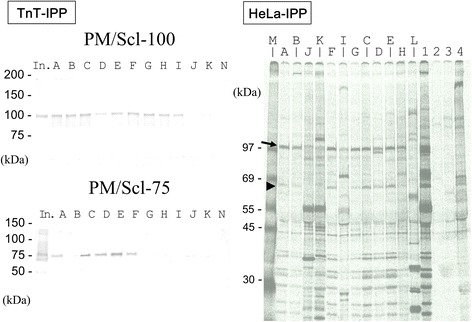
Detection of anti-PM/Scl antibodies in immuoprecipitation analysis. TnT-IPP: immunoprecipitation of biotinylated recombinant PM/Scl-100 and PM/Scl-75. Recombinant proteins were subjected to 4% to 20% SDS-PAGE and analyzed by immunoblotting with streptavidin-alkaline phosphatase and substrate. In., the input was half the dose for immunoprecipitation. Lanes A to K correspond to the anti-PM-Scl-100 and/or −75-positive patients shown in Figure 1. Lane N: healthy control serum. HeLa-IPP: immunoprecipitation analysis using radiolabeled HeLa cell extracts. Lanes A to L correspond to the patients shown in Figure 1 and Table 1. Lanes A to K correspond to anti-PM-Scl-100 and/or −75-positive patients shown in Figure 1. Lane M: [Methyl-^14^C] methylated protein MW markers (PerkinElmer Japan, Yokohama, Japan). Lane L: anti-U1-RNP-positive serum with equivocal titers for both antibodies in ELISA. Lanes 1 to 4 show the reference sera; lane 1, anti-PM/Scl-positive serum; lane 2, anti-MDA5-positive serum; lane 3, anti-TIF1-γ-positive serum; lane 4, anti-Mi-2-positive serum. Arrow and arrowhead corrspond to the PM/Scl-100 and PM/Scl-75 antigens, respectively. IPP, immunoprecipitation; TnT, *in vitro* translation and transcription product.

For the screening of anti-PM/Scl antibodies in large numbers of serum samples, we developed an ELISA system that uses biotinylated recombinant PM/Scl-100 and PM/Scl-75. We screened a total of 600 serum samples obtained from patients with various systemic autoimmune diseases and an additional 36 serum samples from healthy volunteers for both antibodies. Based on the cutoff levels at 5 SDs above the mean value, nine (1.5%) and seven (1.2%) patients were positive for anti-PM/Scl-100 and anti-PM/Scl-75 antibodies, respectively (Figure 1). Five patients (A, C, D, E and F) had both antibodies, four (B, G, H and I) had only anti-PM/Scl-100 antibodies, and two (J and K) had only anti-PM/Scl-75 antibodies. When the cutoff was set at 3 SDs above the mean value, one sample from a patient (L mentioned in Figure 2) with overlap syndrome was just below the cutoff for both antibodies. Subsequently, serum samples from these 12 patients were used for immunoprecipitation to confirm whether they were truly positive for the anti-PM/Scl antibodies. An additional 36 samples from healthy volunteers showed levels below the cutoff for both antibodies.

### Immunoprecipitation using recombinant PM/Scl protein and radiolabeled cellular protein

After the initial screening by ELISA, we investigated antibodies against PM/Scl in sera from 11 anti-PM/Scl-100 and/or anti-PM/Scl-75-positive patients and 1 equivocal patient for their ability to immunoprecipitate biotinylated recombinant PM/Scl-100 and PM/Scl-75 and radiolabeled cellular PM/Scl. All nine anti-PM/Scl-100-positive sera in ELISA immunoprecipitated biotinylated recombinant PM/Scl-100, whereas five of the seven anti-PM/Scl-75-positive sera in ELISA immunoprecipitated biotinylated recombinant PM/Scl-75 (Figure 2, TnT-IPP). Sera that were anti-PM/Scl-75-positive in ELISA but -negative in IPP (J and K) were negative for anti-PM/Scl-100 antibodies in ELISA and IPP. Serum of patient L with equivocal ranges in both ELISAs immunoprecipitated neither recombinant PM/Scl-100 nor PM/Scl-75 (data not shown).

To determine whether the positive sera in ELISA immunoprecipitate the PM/Scl complex, we applied conventional IPP using radiolabeled HeLa cell extract (Figure 2, HeLa-IPP). All nine sera (patients A to I) that had reacted with the recombinant PM/Scl-100 also immunoprecipitated a cellular 100-kDa protein. Eight of these sera also immunoprecipitated a 75-kDa protein, but one another (patient I) did not. Two sera that were positive only for anti-PM/Scl-75 in ELISA (patients J and K) immunoprecipitated neither the 100-kDa nor the 75-kDa protein. The serum from an overlap syndrome patient (L) with an equivocal level of both anti-PM/Scl-100/75 antibodies in ELISA was negative in IPP. According to these results, eight sera (patients A to H) were judged to be positive for anti-PM/Scl antibodies, as was one other serum (patient I), which reacted to recombinant PM/Scl-100 and which immunoprecipitated a 100-kDa cellular protein.

### Indirect immunofluorescence staining patterns of anti-PM/Scl-100 and/or anti-PM/Scl-75-positive sera

In IIF analysis, the eight sera (patients A to H) that immunoprecipitated the 100-kDa and 75-kDa proteins showed nucleolar patterns (Table 1). The serum (patient I) that only immunoprecipitated the 100-kDa protein showed a speckled pattern without nucleolar staining. Two sera (patients J and K) that immunoprecipitated neither the 100-kDa nor the 75-kDa protein, also showed no nucleolar patterns.

**Table 1:** **Connective tissue disease manifestations of anti-PM/Scl-100-ELISA- and/or anti-PM/Scl-75-ELISA-positive patients**

**Patient**	**Age in years**	**Sex M/F**	**Diagnosis**	**IIF pattern** ^**a**^ **, titer**	**ELISA PM/Scl-100/**	**TnT-IPP PM/Scl-100/**	**HeLa-IPP PM/Scl-100/**	**other auto- antibodies**	**clinical features**
					**PM/Scl-75**	**PM/Scl-75**	**PM/Scl-75**		
A	52	F	SS	nucleolar, 1:2560	**+ / +**	**+ / +**	**+ / +**		dry eye, dry mouth
B	62	F	lSSc	nucleolar, 1:320	**+ / -**	**+ / -**	**+ / +**		Raynaud’s ph, sclerodactyly
C	54	M	CADM	nucleolar, 1:640 diffuse, 1:80	**+ / +**	**+ / +**	**+ / +**		ILD, Gottron papules, mechanic’s hands
D	69	M	DM	nucleolar, 1:640 diffuse, 1:80	**+ / +**	**+ / +**	**+ / +**		ILD, Gottron sign, mechanic’s hands, V-neck sign, dysphagia, pharyngeal Ca
E	67	M	DM	nucleolar, 1:1280	**+ / +**	**+ / +**	**+ / +**		ILD, Gottron sign, Heliotrope rash muscle weakness, prostate Ca
F	73	F	UCTD	nucleolar, 1:640	**+ / +**	**+ / +**	**+ / +**		ILD, dry eye, dry mouth
G	33	F	UCTD	nucleolar, 1:640 diffuse, 1:80	**+ / -**	**+ / -**	**+ / +**		morning stiffness, polyarthralgia
H	31	F	UCTD	nucleolar, 1:160 speckled, 1:80	**+ / -**	**+ / -**	**+ / +**		polyarthralgia, photosensitivity
I	31	F	UCTD	speckled, 1:80	**+ / -**	**+ / -**	**+ / -**		oral ulcer, photosensitivity
J	24	F	UCTD	diffuse, 1:640 cytoplasmic, 1:160	**- / +**	**- / -**	**- / -**	SS-A	dry eye, dry mouth
K	23	F	SLE	diffuse, 1:320 cytoplasmic, 1:80	**- / +**	**- / -**	**- / -**	SS-A ribosomal P	polyarthralgia, malar rash, photosensitivity, leukopenia

^a^ ‘diffuse’ and ‘speckled’ in the IIF pattern, respectively, refer to nuclear diffuse and nuclear speckled patterns. CADM, clinically amyopathic DM; Ca, carcinoma; DM, dermatomyositis; ILD, interstitial lung disease; lSSc, limited cutaneous SSc; ph, phenomenon; SLE, systemic lupus erythematosus; SS, Sjögren’s syndrome; SSc, systemic scleroderma; UCTD, undifferentiated connective tissue disease.

### Clinical and laboratory data for anti-PM/Scl-positive patients

The nine patients with anti-PM/Scl were four with UCTD, three with DM (including one with CADM), one with limited cutaneous SSc and one with SS. The clinical features of these patients are summarized in Table 1. The prevalence of anti-PM/Scl in UCTD (25%) is significantly higher than that of DM (2.4%, *P* = 0.0032), SSc (0.5%, *P* = 0.000066), SS (1.2%, *P* = 0.0018), SLE (0%, *P* = 0.00012), OL (0%, *P* = 0.045) and healthy control (0%, *P* = 0.0067). Although the numbers of examined sera are very small, no patients with anti-PM/Scl antibodies are found among patients with PM or OL. Four patients with UCTD are clinically heterogeneous; two are suspected of having SLE, one of having SS and one of having rheumatoid arthritis (RA). All but one are young adult women. No common clinical features, including Raynaud’s phenomenon and abnormal nailfold capillaries, are present among these four patients.

Of the 126 DM patients, there are 8 anti-nucleolar antibody (ANoA)-positive patients, of whom 3 patients, all men, had anti-PM/Scl antibodies. Of the 123 anti-PM/Scl-negative DM patients, only 32 are men (*P* = 0.020). These three patients were complicated with ILD. The clinical manifestations of ILD for these three patients were improved by oral prednisolone and immunosuppressive agent therapy, and their ILD did not have a fatal outcome. Additionally, the complication of internal malignancy (mesopharynx and prostate) was also recognized in two patients three years before or after the disease onset. ILD and internal malignancy are more frequent in anti-PM/Scl-positive DM patients than in anti-PM/Scl-negative DM patients, but not significantly (*P* = 0.060 and *P* = 0.072, respectively).

Besides the three DM patients with ILD, one patient who had UCTD was also complicated with ILD. Although the anti-PM/Scl-100 ELISA units of these four patients with ILD were not higher than those of five anti-PM/Scl-positive patients without ILD (mean 26.7 versus 62.0), anti-PM/Scl-75 titers of the four patients with ILD were significantly higher than those of five patients without ILD (mean 86.0 unit versus 0.96 unit, *P* = 0.027 by Mann–Whitney *U* test).

## Discussion

The anti-PM/Scl antibody is a well-known ANoA and a serological marker of OL and other systemic autoimmune diseases such as SSc, PM and DM alone [15]. This antibody is common in the West. For example, it was the third most-found, followed by anti-centromere and anti-topoisomerase I antibodies, in a large cohort of SSc patients in Germany [31]. However, large studies of Japanese patients with SSc showed this antibody to be absent [32,33], and a recent study noted that 0/588 Japanese patients with SSc had anti-PM/Scl antibodies [18]. Large-cohort studies using sera from more than 200 connective tissue disease patients in the literature are summarized in Table 2, although some studies with mostly overlapped patients are omitted. Anti-PM/Scl antibodies are strongly linked to HLA-DRB1*0301 [34], which is very rarely found in Japanese, with a prevalence of only 0.14%, according to an online database [35] (HLA Laboratory, Kyoto, Japan); however, the contribution of this finding remains unknown. Since we had found three patients to have strong ANoA in IIF analysis during our recent studies on myositis-specific or associated autoantibodies [36-38], we aimed to investigate anti-PM/Scl antibodies in our large cohort of systemic autoimmune disease.

**Table 2:** **Frequencies of anti-PM/Scl antibodies in disease subsets**

**Frequencies of anti-PM/Scl antibodies in disease subsets**						
Study	Marguerie	Mahler	Rozman	Hanke		Maes
Reference	[13]	[39]	[40]	[41]		[42]
Year	1992	2005	2008	2009		2010
Country	UK	Various	Europe	Germany		Belgium
Anti-PM/Scl detection	CIE	PM1-α ELISA	LIA	LIA		PM1-α ELISA
Patient selection and numbers of patients	1689 SLE	205 SSc	625 SSc	280 SSc^b^		70 SSc
	879 SSc^a^	114 SLE		88 RA		66 SLE
	256 PM or DM	40 PM		72 SLE		35 SS
		40 PM/SSc		49 SS		24 RA
						23 DM
						13 PM
						11 MCTD
Anti-PM/Scl-positive patients	27 PM (or DM)/SSc	22 PM/SSc (55%)	1 PM (7.7%)	Anti-PM/Scl-75	Anti-PM/Scl-100	3 SSc (4.3%)
	4 SSc	27 SSc (13%)	18 SSc (2.9%)	29 SSc (10%)	20 SSc (7.1%)	
	1 PM	3 PM (7.5%)	1 DM (1.7%)	3 RA (3.4%)	3 SLE (4.2%)	
				1 SLE (1.4%)	1 SS (2.0%)	
Study	Mierau	Koschik	Mehra	D’Aoust	Kazi	Muro
Reference	[31]	[57]	[58]	[17]	[18]	The present study
Year	2011	2012	2013	2014	2014	
Country	Germany	USA	Australia	Canada	Japan	Japan
Anti-PM/Scl detection	ID	ID	LIA	PM1-α ELISA	IIP	ELISA, IPP
Patient selection and numbers of patients	863 SSc	2425 SSc	528 SSc	763 SSc	Kanazawa cohort	223 SSc
					316 SSc	126 DM
					Keio cohort	123 SLE
					272 SSc	88 SS
						17 overlap
						16 UCTD
						7 PM
Anti-PM/Scl-positive patients	42 SSc (4.9%)	75 SSc (3.1%)	Anti-PM/Scl-75	55 SSc (7.2%)	0	4 UCTD (25%)
			66 SSc (12.5%)			3 DM (2.4%)
			Anti-PM/Scl-100			1 SS (1.1%)
			26 SSc (4.9%)			1 SSc (0.4%)

^a^Since the numbers of myositis overlap patients were not given, the frequencies of the antibodies in disease subsets were not calculated; ^b^51 overlap and 16 undifferentiated connective tissue disease patients were included.

CIE, counter immunoelectrophoresis; DM, dermatomyositis; ID, immunodiffusion; IPP, immunoprecipitation; LIA, line immunoassay; MCTD, mixed connective tissue disease; PM, polymyositis; RA, rheumatoid arthritis; SLE, systemic lupus erythematosus; SS, Sjögren’s syndrome; SSc, systemic scleroderma; UCTD, undifferentiated connective tissue disease.

Although LIA for anti-PM/Scl-75 and −100 antibodies and PM1-α ELISA have often been used recently [16,31,39-43], the latter is not available in Japan and the former is not cost-effective, costing around 13,000 yen/sample (Cosmic Corporation, Tokyo, Japan). For our in-house ELISA, the anti-PM/Scl-75 assay was found to be inferior to the anti-PM/Scl-100 assay both in sensitivity and specificity, according to the results of protein-IPP, which is widely accepted as a reference method for detecting several markers for SSc and PM/DM. Originally, most PM/Scl-positive sera have been shown to contain anti-PM/Scl-100 and about 50% to 60% of the sera have been shown to react with PM/Scl-75 [11,12,44,45]. Raijmakers and colleagues showed that PM/Scl-75 contains a previously unidentified N-terminal region that is important for the antigenicity of the protein [46]. This longer form, named PM/Scl-75c, was as reactive as PM/Scl-100 to sera from PM/SSc overlap patients in ELISA (28% and 25%, respectively) [46]. Subsequently, Hanke and colleagues showed the prevalence of anti-PM/Scl-75c to be higher than that of anti-PM/Scl-100 (10.4% versus 7.1%) in LIA using sera from 280 SSc patients [41]. There are several possible explanations for the lower prevalence of anti-PM/Scl-75 than anti-PM/Scl-100 in this study. The cDNA in this study, PM/Scl-75c-β, has a 17 amino acid insertion at the C-terminus which could introduce conformational changes in epitope [47]. In the study of Hanke and colleagues, recombinant PM/Scl-75 was expressed by a baculovirus [41]. The discrepancies might also be due to racial differences or clinical backgrounds. In a validation study by Jaskowski and colleagues, the anti-PM/Scl-100 LIA had better agreement for the detection of anti-PM/Scl with IPP as the reference method than with PM/Scl-75 LIA and PM1-α ELISA [48].

**Figure 3:**
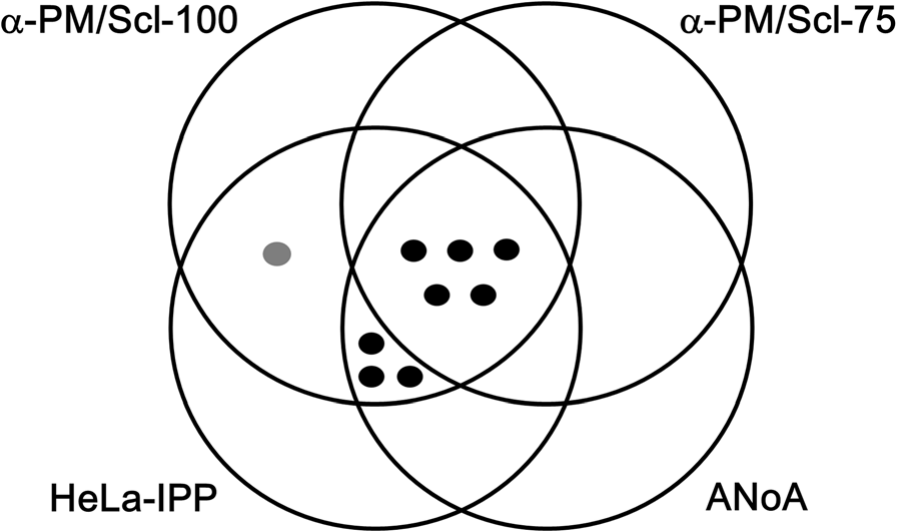
Venn diagram showing the overlap between anti-PM/Scl antibodies by different assays. Anti-PM/Scl-100 and anti-PM/Scl-75 circles show the antibody-positive patiens determined by immunoprecipitation with the corresponding recombinant protein. The HeLa-IPP circle shows anti-PM/Scl-positive patients determined by immunoprecipitation with HeLa cellular protein. Black dots show the patients with both anti-75 kDa and anti-100 kDa antibodies, and the gray dot shows the patient with only anti-75 kDa antibody in HeLa-IPP. The ANoA circle shows the patients demonstrating anti-nucleolar patterns as observed by indirect immunofluorecence.

In this study, eight of nine anti-PM/Scl antibody-positive sera exhibited nucleolar staining in IIF analysis. Some studies have shown that anti-PM/Scl-positive sera do not always demonstrate a nucleolar staining pattern in IIF [16,17,47,49]. Interestingly, one ANoA-negative serum with anti-PM/Scl reacted with PM/Scl-100 but not with PM/Scl-75. Intramolecular epitope spreading from the initial response against PM/Scl-100 to a successive response by other exosomal components has been recognized, as have many other autoantibody responses [50]. Figure 3 shows a four-way Venn diagram depicting the overlap between anti-PM/Scl-100 by TnT-IPP, anti-PM/Scl-75 by TnT-IPP, anti-PM/Scl by cellular IPP and anti-nucleolar pattern by IIF. ANoA-positive anti-PM/Scl antibodies all immunoprecipitated both 100- and 75-kDa proteins in HeLa-IPP, whereas only one ANoA-negative anti-PM/Scl antibody (patient I) immunoprecipitated only a 100-kDa protein in HeLa-IPP. Since sera from Patient I immunoprecipitated several other polypeptides, the nuclear speckled staining of this patient in IIF may correspond to antibodies against these proteins. Moreover, anti-PM1α reactivity has been reported in apparently ANA-negative samples [16,17,49]. Although future studies are necessary to address whether monospecific anti-PM/Scl-100 antibodies show nucleolar staining in IIF, we can conclude that IIF is not a sensitive immunoassay for the detection or screening of anti-PM/Scl antibodies.

The findings of anti-PM/Scl antibodies in UCTD patients are of clinical importance. The classification criteria of UCTD are not well established [51]. Since our UCTD patients were not diagnosed with definite connective tissue disease even if the new set of RA criteria [52] and the preliminary criteria for the very early diagnosis of SSc [53] were used, we applied the preliminary classification criteria of UCTD suggested by Mosca and colleagues [27]. Four UCTD patients with anti-PM/Scl were all so-called ‘stable UCTD’. Their disease courses were stable over a period of more than three years without internal organ involvement, except for one, whose ILD was nonspecific interstitial pneumonia that did not exacerbate for more than ten years. Interestingly, Cordiali-Fei and colleagues [54] reported that anti-PM/Scl responses were mainly associated with Italian patients with UCTD, which was defined by the same criteria used in our study. They found 5 patients with anti-PM/Scl in 23 patients with UCTD (22%), a frequency that is almost the same as that of our study.

Of second importance are the three anti-PM/Scl-positive DM patients. Myalgia or muscle weakness varied, and the levels of creatine kinase ranged from normal levels to more than 2,000 IU/L. Patient C in this study is the second reported case of CADM, to the best of our knowledge, following the first case described by Lega and colleagues [55]. A previous study of 20 PM/DM patients with anti-PM/Scl demonstrated that anti-PM-Scl is not necessarily a marker for good prognosis in patients with PM/DM, because lung and esophageal involvement were found (in 75% and 20%, respectively), as was internal malignancy (in 15%) [56]. Also in our study, all three patients were complicated with ILD and required combined therapy of steroids and immunosuppressive agents. Although they were all alive during the observation periods, the prognosis of ILD in anti-PM/Scl-positive DM patients cannot be determined due to the very limited numbers examined and the limited observation periods (maximum 45 months). Two of these patients were also complicated with localized cancer without metastasis. In a previous study, two out of twelve antibody-positive patients with DM had mechanic’s hands [56]. Very interestingly, also in our study, two patients exhibited mechanic’s hands in addition to sole hyperkeratotic rhagadiform symptoms.

## Conclusions

Our study of Japanese patients with various systemic autoimmune diseases confirms that anti-PM/Scl antibodies also exist in these patients. ELISA with PM/Scl-100 recombinant protein was useful in detecting anti-PM/Scl antibodies. Anti-PM/Scl was not always specific for DM or SSc; it was also present in various autoimmune conditions, including UCTD. All the anti-PM/Scl-positive DM cases were complicated with ILD and/or cancer, while no life-threatening internal organ involvement was found in other anti-PM/Scl-positive cases. Considering the higher prevalence of anti-PM/Scl in UCTD, this autoantibody may be more important in systemic autoimmune disease clinics than we expected. Further studies in larger cohorts are necessary to define the clinical significance of anti-PM/Scl antibodies in Japanese patients with each autoimmune condition. Future collaborative studies for evaluating our sera with LIA and PM-1α ELISA promise to be interesting.

## Abbreviations

ACR: American College of Rheumatology
ANoA: anti-nucleolar antibody
ARS: aminoacyl tRNA synthetase
CADM: clinically amyopathic dermatomyositis
CIE: counter immunoelectrophoresis
DM: dermatomyositis
ELISA: enzyme-linked immunosorbent assay
EULAR: European League Against Rheumatism
ID: immunodiffusion
IIF: indirect immunofluorescence
ILD: interstitial lung disease
IPP: immunoprecipitation
LIA: line immunoassay
OL: overlap syndrome
PM: polymyositis
RA: rheumatoid arthritis
RLU: relative luminescence unit
Scl: scleroderma
SD: standard deviation
SLE: systemic lupus erythematosus
SS: Sjögren’s syndrome
SSc: systemic sclerosis
TnT: *in vitro* translation and transcription product
UCTD: undifferentiated connective tissue disease
